# *Phlebotomus papatasi* SP15: mRNA expression variability and amino acid sequence polymorphisms of field populations

**DOI:** 10.1186/s13071-015-0914-2

**Published:** 2015-05-29

**Authors:** Marcelo Ramalho-Ortigão, Iliano V. Coutinho-Abreu, Valdir Q. Balbino, Carlos Alberto S. Figueiredo, Rami Mukbel, Hussan Dayem, Hanafi A. Hanafi, Shabaan S. El-Hossary, Emad El-Din Y. Fawaz, Mahmoud Abo-Shehada, David F. Hoel, Gwen Stayback, Mariha Wadsworth, Douglas A. Shoue, Jenica Abrudan, Neil F. Lobo, Andrew R. Mahon, Scott J. Emrich, Shaden Kamhawi, Frank H. Collins, Mary Ann McDowell

**Affiliations:** Department of Entomology, Kansas State University, Manhattan, KS 66506 USA; Laboratory of Malaria and Vector Research, NIAID-NIH, 12735 Twinbrook Parkway, Rockville, MD 20852 USA; Department of Genetics, Universidade Federal de Pernambuco, Recife, PE Brazil; Faculty of Veterinary Medicine, Jordan University of Science and Technology, Irbid, 22110 Jordan; Vector Biology Research Program, U.S. Naval Medical Research Unit No. 3 (NAMRU-3), Cairo, Egypt; Department of Biostatistics, Uniformed Services University of the Health Sciences, Bethesda, MD 20814 USA; Eck Institute for Global Health, Department of Biological Sciences, University of Notre Dame, Notre Dame, IN 46556 USA; Department of Pathology and Laboratory Medicine, The Children’s Hospital of Philadelphia, Philadelphia, USA; Department of Biology, Central Michigan University, Mount Pleasant, Detroit, MI 48859 USA; Department of Computer Science and Engineering, University of Notre Dame, Notre Dame, IN 46556 USA

**Keywords:** Sand fly, Saliva, PpSP15, Leishmaniasis, Vaccine, Expression variability, MHC class II epitopes

## Abstract

**Background:**

The *Phlebotomus papatasi* salivary protein PpSP15 was shown to protect mice against *Leishmania major*, suggesting that incorporation of salivary molecules in multi-component vaccines may be a viable strategy for anti-*Leishmania* vaccines.

**Methods:**

Here, we investigated PpSP15 predicted amino acid sequence variability and mRNA profile of *P. papatasi* field populations from the Middle East. In addition, predicted MHC class II T-cell epitopes were obtained and compared to areas of amino acid sequence variability within the secreted protein.

**Results:**

The analysis of *PpSP15* expression from field populations revealed significant intra- and interpopulation variation.. In spite of the variability detected for *P. papatasi* populations, common epitopes for MHC class II binding are still present and may potentially be used to boost the response against *Le. major* infections.

**Conclusions:**

Conserved epitopes of PpSP15 could potentially be used in the development of a salivary gland antigen-based vaccine.

**Electronic supplementary material:**

The online version of this article (doi:10.1186/s13071-015-0914-2) contains supplementary material, which is available to authorized users.

## Background

Hematophagous vectors of disease not only inoculate pathogens into their hosts, these arthropods also dispense pharmacologically active compounds that prevent host homeostasis to facilitate blood feeding. Many of these molecules are immunogenic and elicit host immune responses that reduce the feeding efficiency [1,2] and fecundity [3–8]; furthermore these proteins act as immune effectors that influence the ability of blood-feeding vectors to transmit pathogens [9–11].

Exposure to bites of uninfected *P. papatasi* can lead to protection against *Leishmania major* [12] in murine models. Secreted salivary proteins in *P. papatasi* are estimated to contain between 30 and 35 different protein molecules [13]. One of these molecules, PpSP15, has been studied in detail because it has been shown to confer protection in animals against challenge with *Le. major* [14]. A previous analysis of predicted PpSP15 amino acid sequence polymorphisms involving one field and four laboratory populations of *P. papatasi* indicated the presence of polymorphic alleles and pointed to the possibility of a multi-copy gene family [15]. However, synonymous amino acid substitutions accounted for most of the nucleotide changes identified.

Variability in salivary gland proteins between distinct vector populations may influence disease outcome. Such variability could be represented either by different amounts of one or more salivary proteins being secreted in the saliva and injected in the host, or by differences in amino acid sequence. In regards to the latter possibility, amino acid sequence variability may influence class II MHC presentation of the salivary antigen, inducing different immune responses by the host. For PpSP15, 40 alleles have previously been identified, most of which are geographically restricted [15]. However, the PpSP15 alleles differ by an average of 1.7 %, with most differences being synonymous. This observation suggests that this gene is not under diversifying selection and is not surprising given the relative homogeneity of *P. papatasi* from distant geographical locations [16–18].

Here we assess the variability of PpSP15 from field collected populations of female *P. papatasi.* Collection locations for this investigation include locations in the Middle Eastern regions of Southern Egypt, the Northern Sinai Peninsula, and Jordan. Using molecular genetic tools, we elucidated differences in mRNA expression levels, amino acid variability, and genetic differentiation. Our results suggest a level of variability that is compatible with geographically isolated populations, and more importantly higher than previously reported. Additionally, *PpSP15* expression levels are differentially modulated in different ecological habitats. Our findings further suggest that despite the level of variability detected for predicted human MHC Class II peptides of PpSP15, conserved epitopes are present and could potentially be used in the development of a salivary gland antigen-based vaccine or natural adjuvant, possibly utilizing PpSP15 and/or a combination of PpSP15 and other salivary proteins.

## Methods

### Sand flies

*P. papatasi* used in this study were obtained either from field collections or from a colony (Israeli strain - PPIS) maintained at the University of Notre Dame. This PPIS colony is from a colony originally established in the mid 1970’s and represents limited genetic heterogeneity. For field samples, sand flies were collected at 4 locations, 2 in Egypt and 2 in Jordan. 1) Aswan (GPS coordinates N 24°10, E 32°52), in a village adjacent to the River Nile (Baharif - Southern Egypt); this site was chosen because *P. papatasi* is prevalent but leishmaniasis is absent, the village is cultivated with clover (*Trifolium* spp.), corn (*Zea mays*), wheat (*Triticumaestivum*), date palms (*Phoenix dactylifera*) and mangoes (*Mangiferaindica*), and stocked with a variety of domestic animals [19]. 2) Northern Sinai (GPS coordinates N 30°50′, E 34°10′), in a Bedouin village (Om Shikhan – Northeastern Egypt); this site is a typical rolling sand desert with an uncharacteristically heightened water table with uncultivated areas covered by low desert brush, predominatly *Artmeisia, Salicornia, Thymelaea, Tamarisk* and *Panicum.* The area is endemic for *L. major* infections [20], however the typical rodent reservoir host *Psammomys obesus*is limited and *Gerbillus pyramidum* serves as the primary reservoir in this area [21]. 3) Swaymeh (GPS coordinates N 31°48′, E 35°35′), near the Dead Sea, in Jordan; this site, endemic for zoonotic *Le. major*, maintained by the reservoir *Ps. obesus* [22–25]. The location is considered Saharan Mediterranean with a mean rainfall of <50 mm occurring only November –April and has tropical and halophytic vegetation (chenopods) [26]. 4) Malka, near the Northern border of Jordan (GPS coordinates N 32°40′, E 35°45′). The biotope at this site is more Mediterranean and the land is rocky. At the time these flies were collected only *Le. tropica* infections had been reported, thought to be due to the absence of the rodent reservoir *Ps. obesus* [27].

Whenever possible, sand fly trappings were carried out three times a year, early (June), middle (August) and late (September) for years 2006 and 2007. While in Aswan and Swaymeh we performed 3 trappings (late 2006, early and middle 2007); for the site in North Sinai only 2 trappings took place: early and middle 2007, due to security concerns in 2006. For each of these 3 locations *P. papatasi* represents approximately 95 % of the *Phlebotomus* species [19,24]. In Malka, trapping took place in late (September) 2006. Sand flies were trapped using CDC-style light traps between 18:00 and 06:00. Traps were either baited with CO_2_ (dry ice) (for trappings done in Aswan and North Sinai), or non-baited (Swaymeh and Malka). Sand flies were transferred from collection bags and maintained alive until dissected. Flies were euthanized in water and detergent just prior to dissection. *P. papatasi* were identified by microscopic examination of female spermateca according to Lane [28].

### Sample preparation

Sand flies were assessed for their blood fed status and parity. For flies without the presence of blood, ovaries were examined and parous were separated by non-parous flies by the presence of granulous in the accessory glands [29]; only non-parous flies were used in our analysis. Heads with both salivary glands from *P. papatasi* females were separated from the bodies under a dissecting microscope and placed in 1.5 ml microcentrifuge tubes containing 50 μl RNA later (Ambion) and immediately homogenized using a hand held homogenizer RNAse-free pestle. Samples were stored at 4 °C for up to 48 h, frozen in dry ice for transport, and kept at −80 °C until further analyzes. For sequence variability analyses, 50 individuals from each of the three field populations were used whereas for expression profile comparisons, mRNA (cDNA) levels of 20 specimens collected per time point per population were assessed. The sand flies used were representatives of the populations from Aswan (or PPAW), North Sinai (or PPNS), Malka (or PPJM), and Swaymeh (or PPJS).

### RNA extraction and cDNA synthesis

Total RNA was isolated from individual sand fly samples using the RNeasy Mini RNA isolation kit (Qiagen). Total RNA was extracted from the dissected heads with salivary glands from field and laboratory (PPIS) sand flies. For each RNA sample, PpSP15 cDNA was synthesized using Invitrogen reagents and following the manufacturer’s instructions. In brief, 12 μl RNA was added with 2.5 μM Oligo (dT)_20_ primer and 0.5 μM dNTPs (10 mM), incubated at 65 °C for 5 min (min) and kept in ice for at least 1 min; 4 μl 5X SuperScript™ III Reverse Transcriptase First-Strand Buffer, 5 mM DTT (0.1 M), 20 Units of RNase OUT, and 200 units of SuperScript™ III Reverse Transcriptase (200 units/μl) were added to the reaction. The mixture was incubated for one hour at 50 °C and stored at −20 °C.

### Real-time quantitative polymerase chain reactions (RT-qPCR)

RT-qPCR reactions were set up with 10 μl SYBR Green reagent (Applied Biosystems), 0.6 μl each forward and reverse primer (0.3 μM final concentration), 0.5 μl each cDNA sample, and 8.3 μl Ultra Pure DNase/RNase-Free Water (Invitrogen). Reactions were analyzed in 96-well plate format using a 7900HT Fast Real Time PCR System (Applied Biosystems) under the following conditions: initial incubation at 50 °C for 2 min (min) and 95 °C for 10 min; followed by 40 cycles of 95 °C for 15 s (sec), 55 °C for 1 min; ending with a dissociation step of 95 °C for 15 s, 55 °C for 15 s, and 95 °C for 15 s.

Twenty samples from the field populations PPAW, PPNS and PPJS were analyzed by RT-qPCR. For qPCR, a 152 bp fragment of the PpSP15 cDNA was amplified using PpSP15 specific primers SP15_152F 5′- GGACAAAAGCCTGAAAGCAG and SP15_152R 5′- GAGGTCCAATTCGTTTGTCG. As control mRNA to the q PCR reactions primers PpTub-P24F and PpTub-P24R that amplify a 247 bp fragment of the α-tubulin gene in *P. papatasi* were utilized [30].

Differential expression results for each salivary protein gene were displayed as fold changes over a control, using the 2^-ΔΔCT^ method [31]. The fold changes were calculated by the expression 2^-ΔΔCT^, where ΔΔC_T_ = ΔC_T_(sample) – ΔC_T_(calibrator), ΔC_T_ = ΔC_T_(sample) – ΔC_T_(alpha tubulin gene), C_T_ = cycle at which a statistically significant increase in the emission intensity over the background. The calibrator was represented by the average expression (mean ΔC_T_) of seven non-fed samples (PPIS) dissected 24 h after emerging [32]. Fold changes were calculated for each sample, compared to the calibrator.

### Expression profiling and statistical analysis

**Fig. 1:**
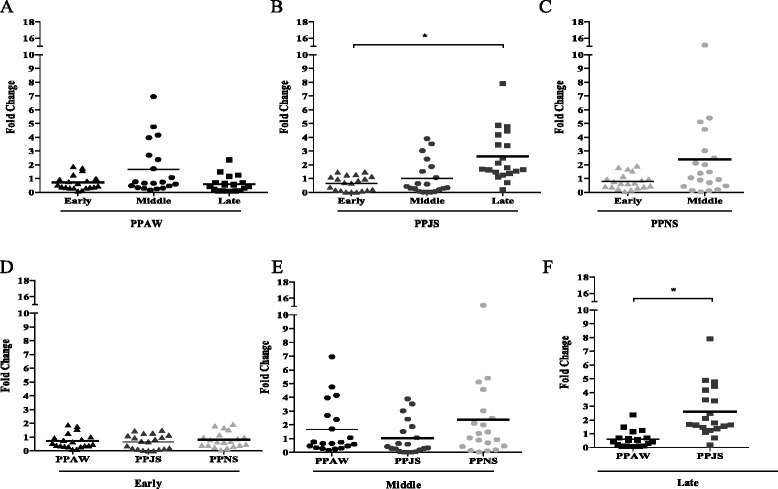
*PpSP15* expression. Expression profiles by real time PCR were assessed as fold changes (Y axis) over the control non-sugar fed, and colony-maintained *P. papatasi* using the 2^-ΔΔCT^ method. Seasonal analyses are displayed in (**a**), (**b**) and (**c**), representing the populations of Aswan (PPAW), Swaymeh (PPJS) and North Sinai (PPNS), respectively. *P. papatasi* were collected at different periods during the sand fly activity season. Graphs (**d**), (**e**) and (**f**) display the geographic comparisons between expression profiles of *P. papatasi* collected early (June) and in the middle (August) of the 2007 season, and late (September) in the 2006 season, respectively. Horizontal bars represent the expression mean values between the samples and each sample represents an individual fly. Asterisk (*) indicates statistically significant differences (*p* < 0.0006) between every two groups analyzed. Aswan (PPAW, black), Swaymeh (PPJS, dark gray) and North Sinai (PPNS, light gray) color schemes are shown. Triangle, circle and square represent expression levels of sand flies collected early, in the middle and late in the season, respectively

Two types of analyses were carried out to compare *PpSP15* expression profiles. For the seasonal analysis, expression levels for each of the 20 sand flies collected at different time points of the season (Fig. 1a-c) in each habitat were compared (Fig. 1d-f). For the geographic analysis, we compared individual expression levels from flies collected in different geographic locations but at the same seasonal period.

Statistical analyses were carried out using the software GraphPad Prism v. 5.01 (GraphPad Software, Inc). Non-parametric Kruskal-Wallis tests were performed to compare between the multiple datasets, and/or Mann–Whitney tests, for 2-way comparisons between data sets when the results for Kruskal-Wallis test were statistically significant, or for comparisons when only two data sets were present. The level of significance was adjusted for multiple comparisons using Bonferroni’s correction to allow comparison of the PpSP15 expression profile with the pattern displayed by nine other *P. papatasi* salivary transcripts [33]. Differences were considered statistically significant at α = 0.0006. Values of fold change were calculated based on the ratio of the expression medians between the time point up-regulated over the time point down-regulated.

### Sequence analyses

cDNAs produced from the total RNA obtained from PPAW, PPJS and PPJM sand flies were amplified by PCR using primers SP15F 5′-TTAATTTCCGCAGTGTTC-3′ and SP15R 5′- TTATATATTGATTGTTTTATC-3′ and GoTaq (Promega) with the 2x mastermix. The 408 bp *PpSP15* mature cDNA PCR products were twice washed in 150μlDNAse/RNAse-free water (Invitrogen) using Multiscreen PCR cleaning plates (Millipore) and by applying vacuum (10 psi). Purified PCR products were resuspended in 50 μl sterile water and cycle-sequenced on an ABI3730XL using two separate sets of overlapping primers. One set included the primers PpSP15_152 forward and PpSP15_152 reverse shown above while the second set included the primers PpSP15_206F 5′-CTGCCAAGCTGAAAATCCAT-3′ and PpSP15_206R 5′-GCTTTCAGGCTTTTG-3.

Both leading and lagging strands of all PCR products were sequenced twice and only resulting sequences with Phred values [34] with a cut off ≥ 30 were used in the further analyses. The forward and reverse chromatograms were inspected and manually corrected using the Staden Package [35] and sequences from each specimen were grouped using the program Cap3 [36]. Sequence alignments were performed using an individual organism’s consensus sequences using ClustalW [37] using basic parameters and the aligned datasets were visualized with Mega v. 4.1 [38]. The resulting sequences were deposited in GenBank (http://ncbi.nlm.nih.gov) under accession numbers HM470028 to HM470100.

### *Cloning of* PpSP15

High fidelity Taq (PrimeSTAR HS, Takara/Contech) was used to amplify the SP15 sequence from cDNA using the primer set: SP15forII-GGACTTGCTTTAATTTCCGCAGTGT and SP15rev-TTATATATTGATTGTTTTATCATA. Amplicons were cloned using Zero Blunt TOPO PCR cloining kit (Invitrogen). At least 10 clones were sequenced from each individual. Sequences were trimmed, aligned and SNPs analyzed using Geneious software [39].

### Read depth analysis

Source read data used for the newly assembled *P. papatasi* genome were first aligned using BWA version 0.5.9-r16 against the resulting scaffolds made available from VectorBase (www.vectorbase.org). A custom perl script was used to determine coverage at each position of the genome using CIGAR-formatted alignments derived from the BWA-produced SAM file. Based on these positional coverage data, mean coverage and the global standard deviation were computed. Next, a local mean and standard deviation were computed in a separate Perl script for each putative exonic sequence determined by BLAST and used to compute a Welch t-statistic, which can access statistically significant differences in means with unequal samples and variances, typical of such window-based analysis. P-values for higher (or lower) than expected coverage was finally computed using these statistics (one per exon) in Microsoft Excel.

### Population analyses

For population assessments, analyses were initially performed at the intra-population level where sequence polymorphism from all three populations was assessed separately for the following parameters: nucleotide diversity (Pi); number of segregating sites (S), and haplotypic diversity (Hd), as described in [40]. Further analyses were performed at the inter-population level to determine Hs and Ks indexes [41], and the fixation indexes Fst [42], Gst [43] and Nst [44]; Genetic distances were calculated based on the Jukes and Cantor [45] correction. The neutral evolution hypothesis [46], as well as the neutrality tests Tajima’s D [47], and Fu & Li’s D* and F [48], and Fs [49] also were assessed. Da/Ds ratio (ω) was performed for the entire SP15 sequence as well as for slide windows of 15 codons each. All parameters above were calculated using DnaSP v.5 [50].

The relative frequencies of polymorphic nucleotides and amino acid residues were graphically represent in the form of weblogos [51]. A weblogo is a graphical representation of a multiple sequence alignment in which the height of the bases in each position indicates their relative frequency, whereas the overall weight of the stack indicates the conservation of that position in the motif. The relationships among haplotypes were obtained by the median joining method using Network 4.5.1.6 (www.fluxus-engineering.com).

### Secondary structure and T-cell epitope predictions

For prediction of the secondary structure, we first produced a consensus sequence using all of the predicted amino acid sequences for SP15 using Bioedit v.7.0.9 [52]. The consensus sequenced obtained (using the majority rule) was applied to the secondary structure prediction tool (http://bioinf.cs.ucl.ac.uk/psipred/) using default parameters.

To identify predicted promiscuous HLA-class II binding sites and human T-cell epitopes, we used three prediction tools. The TEPITOPE software package [53], the IEDB Analysis Resource T cell Epitope Prediction Tools (http://tools.immuneepitope.org/main/html/tcell_tools.html), and the ProPred MHC class II binding prediction server (http://www.imtech.res.in/raghava/propred/) [54]. Threshold for promiscuous search was set a 3 % and search was performed for 25 different HLA-DR for TEPITOPE and 51 HLA-DR for ProPred. For the 65 HLA and 2 H2 from the IEDB, only predicted peptides with SMM_align or COMBLIB scores of 1000000.00 are included.

### Ethical approval

The study protocol was approved by the Naval Medical Research Unit No. 3 Institutional Review Board (IRB) in compliance with all applicable Federal regulations governing the protection of human subjects. IRB # 193, DoD # NAMRU3.2006.0011 and the IRB at the University of Notre Dame (#08-135). In addition, the study protocol was approved by the Institutional Animal Care and Use Committee at the University of Notre Dame (#07-052).

## Results

### PpSP15 expression levels in field populations

Expression level analyses of SP15 mRNA were performed on three geographically distinct populations of *P. papatasi* from Aswan (PPAW) and North Sinai (PPNS) in Egypt, and Swaymeh (PPJS) in Jordan. The results from the real time PCR, performed on 20 individual sand flies per collection per population, indicate that PPAW and PPNS display similar expression level for *PpSP15* (Fig. 1). The data also suggest seasonality in the expression levels of *PpSP15* for PPJS. For PPAW individuals, no statistically significant differences were detected regarding seasonal analysis (Fig. 1a). On the other hand, PPJS samples displayed a 2.5 fold greater expression towards the end of the sand fly activity season (Fig. 1b). Although no statistical significant seasonal variation was detected for PPNS (Fig. 1c), we were unable to gain access to the PPNS site during the late season time point.

Early in the season (June), *PpSP15* mRNA expression levels were similar between all three populations, with a maximum of two-fold difference between the lowest and the highest samples (Fig. 1d). For trappings made in the middle of the season, a greater variability was detected (Fig. 1e), although differences in expression levels between natural populations were not statistically significant. Late in the season, however, differences in expression levels between PPAW and PPJS were statistically significant (*p* < 0.0006) with a median difference of 3.6 fold (Fig. 1f).

### cDNA analyses and haplotype identification

**Fig. 2:**
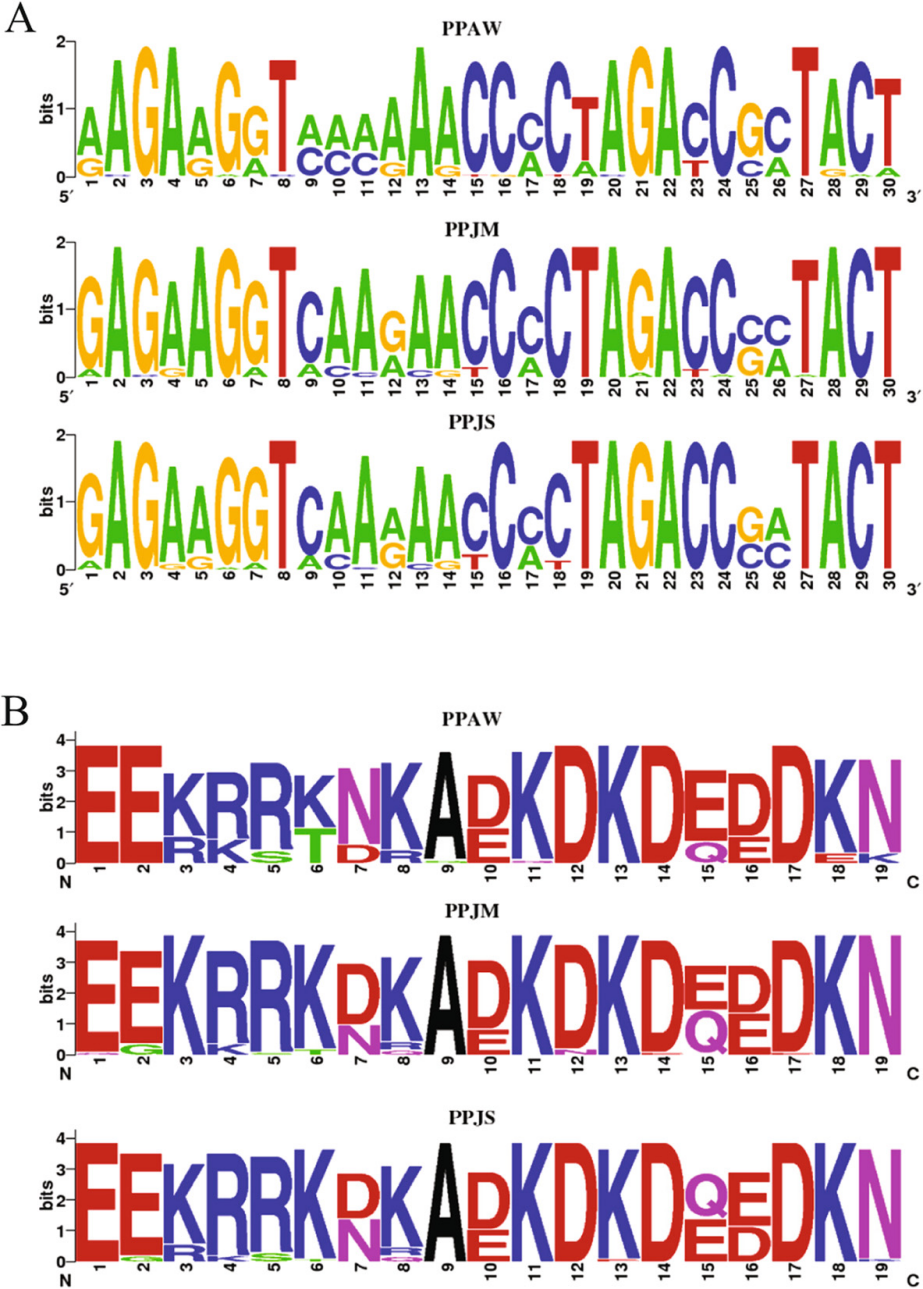
PpSP15 variability.**a** Weblogo representing the relative frequencies of the nucleic acid polymorphic sites found for *PpSP15* in the natural *P. papatasi* populations of PPAW, PPJM, and PPJS. **b** Weblogo representing the relative frequencies of the amino acid polymorphic sites found for the predicted PpSP15 in the natural *P. papatasi*populations of PPAW, PPJM, and PPJS

Genetic variability was assessed in three geographically distinct populations of *P. papatasi* from Aswan (PPAW) in Egypt, and Swaymeh (PPJS) and Malka (PPJM) in Jordan. Following amplification of the PpSP15 mature cDNA from field collected populations (PPAW, PPJM, and PPJS) and colonized *P. papatasi*, the 408 bp PCR fragment from the PpSP15 gene was sequenced with the overlapping primer sets PpSP15_152 F and R, and PpSP_206 F and R. Sequences were then assembled, screened and corrected using a Phred cutoff value of 30, resulting in a total of 99 high quality sequences from PPAW (n = 30), PPJM (n = 36) and PPJS (n = 33). From the resulting sequences, 73 unique haplotypes were identified with 66 unique to a single geographic location and six shared between sites. Additional file 1: Table S1 identifies haplotypes, their frequencies and distributions, and the corresponding predicted SP15 peptides. In spite of considerable genetic homogeneity between the populations, significant differences in the frequency of nucleotides for several sites were identified (e.g., positions 9, 10, and 11 - nucleotides A/C – that display distinct differences between the two Jordanian *P. papatasi* populations (PPJM and PPJS) and Egypt (PPAW (Fig. 2a). The resulting peptides revealed a substantial level of conservancy between the different populations, however (Fig. 2b). Seven of the 50 peptides identified were shared between the PPJM and PPJS populations (these two populations provided the majority of the shared peptides identified). Only two peptides (PPSP1503 and PPSP1505) were distributed among the three populations, reinforcing the idea that the level of genetic similarity between the Jordanian populations (PPJM and PPJS) is greater than the genetic similarity between any of the two Jordanian populations studied and PPAW.

### DNA sequence and population genetics analyses

**Fig. 3:**
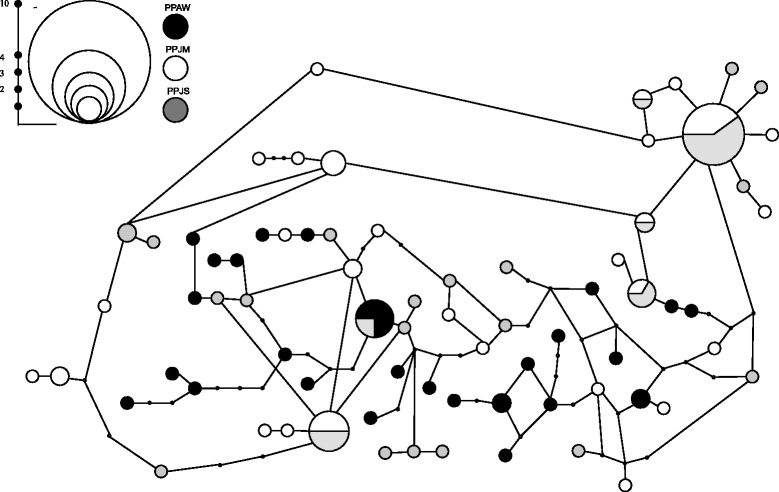
Parsimony network produced from DNA sequence data of PpSP15 using TCS 1.21 [73]. Connection limits of 95 % were used between haplotypes and gaps were treated as missing data. Reticulations in the resulting network were broken using the rules of Crandall *et al*. [74]. Circle diameter is representative of haplotype frequency and color representative of collection location as denoted in the legend and scale bar

**Fig. 4:**
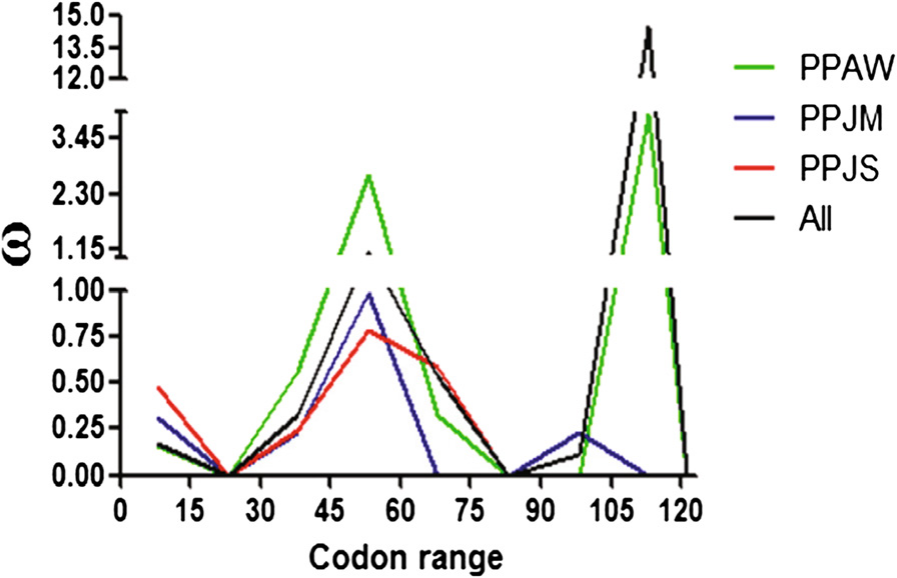
Sliding window analysis. Omega values (ω) were plotted for every 15 codons in the PpSP15 sequence of PPAW, PPJS, PPJM, and for all the sequences together in a single group (All). The last window (9^th^) presented a single codon. Values above one are indicative of positive selection

Population-level analyses were performed on 99 high quality (Phred/Phrap 30 cut off) cDNA sequences of PpSP15 obtained from *P. papatasi* samples. The assessments of genetic diversity including monomorphic and polymorphic, single variable, parsimony, and segregating sites were also determined (Table 1). Little to no population structure was identified between the populations (Table 2); however, the resulting haplotype network (Fig. 3) revealed a closer relationship between the two Jordanian populations. Furthermore, we observed ω values (da/ds ratio) that are suggestive of diversifying selection for at least portions of the molecule. Omega (ω) values higher than one were detected for PPAW, as well as when all the sequences where assessed in a single group, between amino acids residues 46–60 and 106 – 121 (Fig. 4).

**Table 1:** Summary of the genetic/population parameters assessed for *P. papatasi* populations from Egypt and Jordan

Population	PPAW	PPJM	PPJS	ALLDATA
Number of sequences	30	36	33	99
Number of sites	369	369	369	369
Monomorphic sites	346	351	351	339
Polymorphic sites	23	18	18	30
Singleton variable sites	8	3	4	9
Site positions	12 48 129 178 185 207 217	22 330 351	48 134 304 363	12 22 129 185 217 304 330 351 360
Parsimony informative sites	15	15	14	21
Site positions	9 29 119 130 132 134 142 173 189 216 312 340 345 352 363	9 26 119 130 132 134 142 172 173 178 189 301 312 340 345	9 26 29 119 130 132 142 172 173 178 189 207 340 345	9 26 29 48 119 130 132 134 142 172 173 178 189 207 216 301 312 340 345 352 363
Segregating sites (S)	23	18	18	30
Total number of mutations (Eta)	23	18	18	30
Total number of synonymous changes	10	5	5	11
Site positions	9 12 48 129 132 178 207 216 312 360	9 132 172 178 312	9 48 132 178 207	9 12 48 129 132 172 178 207 216 312 360
Total number of replacement changes	13	13	13	19
Site positions	29 119 130 134 142 173 185 189 217 340 345 352 363	22 26 119 130 134 142 173 189 301 330 340 345 351	26 29 119 130 134 142 172 173 189 304 340 345 363	22 26 29 119 130 134 142 173 185 189 217 301 304 330 340 345 351 352 363
Number of haplotypes	26	28	25	73
Haplotype diversity (Hd)	0.989	0.981	0.966	0.986
Standard Deviation of Hd	0.013	0.013	0.022	0.006
Nucleotide diversity (*Pi*)	0.017	0.011	0.012	0.014
Standard deviation of *Pi*	0.00094	0.00097	0.00085	0.00063
Theta (per site) from Eta	0.01573	0.01176	0.01202	0.01573
Theta (per site) from S (Theta-W)	0.01573	0.01176	0.01202	0.01573
Standard deviation of theta (no recombination)	0.0057	0.0044	0.0045	0.0047
Standard deviation of theta (free recombination)	0.0033	0.0028	0.0028	0.0029
Theta (per site) from Pi	0.0174	0.0111	0.0118	0.0145
Average number of nucleotide differences (k)	6.274	4.049	4.292	5.225
Theta estimated from Eta	5.806	4.341	4.435	5.806
Fu and Li’s D test statistic	−0.5533	0.5303	0.2017	−1.0383
Statistical significance	NS (*P* > 0.10)	NS (*P* > 0.10)	NS (*P* > 0.10)	NS (*P* > 0.10)
Fu and Li’s F test statistic	−0.3350	0.3347	0.1194	−0.8941
Statistical significance	NS (*P* > 0.10)	NS (*P* > 0.10)	NS (*P* > 0.10)	NS (*P* > 0.10)
Tajima’s D	0.2848	−0.2235	−0.1094	−0.2892
Statistical significance	NS (*P* > 0.10)	NS (*P* > 0.10)	NS (*P* > 0.10)	NS (*P* > 0.10)
Synonymous sites Tajima’s D(Syn)	−0.6029	−0.6167	−0.4688	−0.7157
Statistical significance	NS (*P* > 0.10)	NS (*P* > 0.10)	NS (*P* > 0.10)	NS (*P* > 0.10)
Non synonymous sites Tajima’s D(NonSyn)	0.9544	−0.0065	0.0742	0.0250
Statistical significance	NS (*P* > 0.10)	NS (*P* > 0.10)	NS (*P* > 0.10)	NS (*P* > 0.10)
Silent sites Tajima’s D(Sil)	−0.6029	−0.6167	−0.4688	−0.7157
Statistical significance	NS (*P* > 0.10)	NS (*P* > 0.10)	NS (*P* > 0.10)	NS (*P* > 0.10)
Tajima’s D (NonSyn/Syn) ratio	−0.1583	0.0105	−0.1582	−0.0349
Ω (Da/Ds)	0.512	0.797	0.747	0.565

**Table 2:** The *Phlebotomus papatasi* populations studied

POP 1	POP 2	Hs	Ks	Gst	Fst	Dxy	Da
PPAW	PPJM	0.98436	5.06028	0.00776	0.17662	0.01699	0.00300
PPAW	PPJS	0.97663	5.23543	0.01003	0.15960	0.01703	0.00272
PPJM	PPJS	0.97378	4.16517	−0.00016	−0.00070	0.01129	−0.00001

Populations: PPAW, Aswan; PPJM, Malka; PPJS, Swaymeh. The indexes Hs, Ks, Gst, Nst, Fst, Dxy e Da correspond to: Hs, average of haplotipic diversity between the populations; Ks, average of nucleotide diversity for each population; Gst and Fst, fixation indexes estimated from the haplotipic diversity; Dxy, average nucleotide substitution per site; Da, number of substitutions per site

### Copy number assessment

Previous work indicated that *PpSP15* was a multi-copy gene [15]. As the presence of more than one gene copy violates the assumptions of most mathematical tests of selection, we assessed the *PpSP15* locus of the newly assembled *P. papatasi* genome available from VectorBase (www.vectorbase.org). According to the assembled genome, there is only one copy of *PpSP15*. We hypothesized that assembly issues corresponding to the *PpSP15* locus could have atypical coverage because of allele collapse (multiple genes assembling into a single locus with high coverage). To test this possibility, the source read data used for the assembled *P. papatasi* genome were first aligned against the scaffolds and the mean read depth for the *PpSP15* locus was calculated. None of the three scaffolds (5990, *p* = 0.081; 11004, *p* = 0.073; and 53716, *p* = 0.081) that contain parts of the *PpSP15* coding sequence exhibited a significantly higher read depth that would be expected if more than one copy of *PpSP15* was collapsed by the assembly process into a single locus.

To further investigate the possibility that *PpSP15* is a multi-copy gene, we cloned *PpSP15* from 17 individual female *P. papatasi* collected from the 3 locations (PPJM-5; PPJS-6; and PPAW-6) and sequenced 10 clones from each individual. From this analysis we identified 16 different haplotypes, 10 that were new compared to the PCR sequencing analysis (Additional file 2: Table S2). Of the 10 new haplotypes, however, only 2 were identified from more than 1 individual. Seven individuals expressed 3 alleles, indicating a multi-copy locus. In all cases where 3 alleles appeared to be present, the 3^rd^ allele was found only once from the 10 clones sequenced from that individual. In addition, in all cases except for one (PPSP1504), the 3^rd^ allele was only found once out of the total of 170 sequenced clones. The PPSP1504 allele was found a total of 31 times from 5 different individuals.

### Secondary structure and T-cell epitope predictions

Of the nine variants of *PpSP15* shared by at least two specimens in our study (2 % frequency) that display the previously identified polymorphic sites [15], the majority of these polymorphisms occur within predicted α-helices (sites K40, R44, T45, N48, K58, and D63) and only one within a β-strand (K25). With the exception of K25, all the other previously identified polymorphic sites in the mature protein were confirmed in our study. Nine (E8, E9, K10, A62, K73, D110, Q114, E115, D117) of the 13 novel polymorphic sites we identified are within an alpha helix and four (D101, K102 K118, and N121) are within coils.

**Fig. 5:**
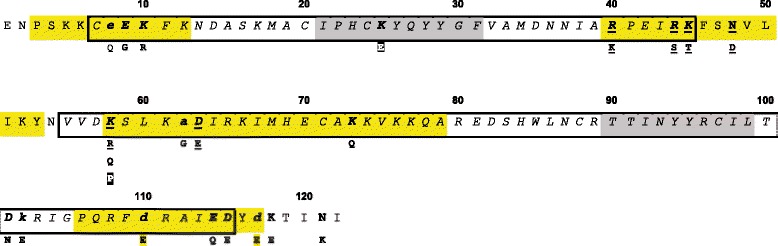
PpSP15 polymorphisms. The predicted PpSP15 mature amino acid sequence is shown (based on sequence accession # AAL11047). Lower case characters represent singletons (unique polymorphic sites) identified in this study; In bold are shared polymorphic sites found in our analyses; bold and underlined are polymorphic sites present in our analyses and also identified by [12];black box characters represent shared polymorphisms found only by [12]. Yellow, gray and white background segments represent alpha helices, beta sheets and coil, according to secondary structure prediction. Regions of PpSP15 displaying predicted MHC class II peptides are boxed

Multiple promiscuous MHC class II epitopes were identified using the three predictive software tools (Fig. 5). Using the threshold setting of 3 (on a scale 1-to-10, 1 = most stringent; 10 = least stringent), the number of predicted epitopes varied according to HLA allele, ranging from none predicted (such as in the case of alleles DRB1_ 1104, DRB1_ 1106, and DRB1_ 1311) to as many as 7 predicted binding peptides (as in the case of alleles DRB1_0305, DRB1_0306, DRB1_0307, and DRB1_0311). The majority of the predicted MHC class II binding sites (17 out of 22 epitopes from 67 alleles) were localized between the isoleucine residue at position 43 (I43) and the glutamine at position 107 (Q107) in the secreted PpSP15 sequence. Many of the binding sites in this region of the protein are overlapping. Four of the MHC class II epitopes predicted were found between tyrosine 28 (Y28) and alanine 39 (A39), and a single predicted MHC class II epitope was predicted between phenylalanine 11 (F11) and alanine 19 (A19).

Interestingly, the region from I43 to Q107 also has the majority of the polymorphic amino acid sites – nine (Fig. 5). Out of the seven polymorphic previously identified sites [15], five are found between amino acids I43 and Q107. These polymorphic sites were confirmed in our analyses. Of the novel 13 polymorphic sites identified in this study, three (E8, E9, K10) located towards the N-terminus portion of the secreted PpSP15 are within a region with the fewest predicted MHC class II epitopes (not shown). Four polymorphic sites (E115, D117, K118, and N121), located towards the C-terminus of PpSP15, were not predicted to be part of any MHC II binding epitopes. Finally, D110 was present within a single predicted MHC II epitope.

The analyses of the PPAW, PPJM and PPJS populations led to the identification of 19 polymorphic sites throughout the mature PpSP15, including the majority of sites described by Elnaiem *et al.* [15] (shown in Fig. 5). All polymorphic sites, with the exception of one in position 48 (K48P) displayed amino acid substitutions considered chemically similar according to the BLOSUM matrix described [18].

## Discussion

Sand fly saliva or salivary components incorporated into multi-component vaccines may be a viable strategy in developing vaccines to combat leishmaniasis. However, if sand fly saliva as a vaccine component is to be successful, an understanding of the genetic variability of salivary proteins in sand fly populations that would be encountered in nature and the human immune responses to such variability is of paramount importance. In this study, we detected significant intrapopulation seasonal variability in expression levels of *PpSP15*, as well as genetic variability between populations and weak genetic structure within and between distant genetic localities in the Middle East.

Previous work indicates that salivary components vary between different field populations [15,33,55]. Furthermore, sand fly saliva variability associated with natural populations was shown to impact the vertebrate response and leishmanial disease progression in a manner significantly different than what was observed for laboratory reared sand flies [56–58]. PpSP15 is among the best studied sand fly salivary proteins and was shown to induce a strong delayed-type hypersensitivity (DTH) response that is sufficient to protect animals against *Le. major* infection [14]. Previous studies have reported low levels of *PpSP15* variation [15]. Evidence also suggests that *PpSP15* is a multicopy gene [15] and SP15 may in fact represent a family of proteins that are highly conserved between sand flies from distant locations [59]. Our results indicate intra- and interpopulation variability of *PpSP15* for the two parameters investigated (i.e., predicted amino acid variability and mRNA abundance). Although in many instances the variability was associated with synonymous substitutions, a considerable number of non-synonymous substitutions were also detected.

For *PpSP15* mRNA expression levels, intra-population differences were detected especially for sand flies collected later in the trapping season (Fig. 1a-c). These differences may be due to the physiological state of the individuals, the source of the blood meal an individual might have taken, or they may be associated with a genetic advantage. Sand fly age and diet was recently shown to be determinant factors on salivary composition in the New World sand fly *Lutzomyia longipalpis* [60]. In addition, *P. papatasi* midgut enzymes are affected by different habitats, whereas oasis sand flies displayed higher chitinolytic activities, flies from arid environment displayed greater glycosidase activity [61]. However, desert flies also exhibit significant differences in glycolytic activities between the spring (flowering season) and fall (end of dry season) [61], pointing to seasonal influences in protein production by sand flies. This study found that *PpSP15* expression is significantly up-regulated late in the season in sand flies from dryer habitats (PPJS from Swaymeh, Fig. 1b), and in significantly higher levels when compared to *P. papatasi* from irrigated areas, such as PPAW from Aswan (Fig. 1f), similar to what we observed for other salivary gland genes [33]. In irrigated regions, fly longevity is increased compared to arid regions [62,63], indicating that age variation and associated body size might explain the differences we detected in *PpSP15* expression late in the season. However, *PpSP15* was not consistently regulated by age and diet in a colonized *P. papatasi* population [64], suggesting that other unknown abiotic factors may play a significant part in driving *PpSP15* expression in natural sand fly populations. While we did not assess sand fly size in this study, starvation has been shown to increase body size [26], that could influence salivary gland gene expression.

The complete PpSP15 precursor (Genebank accession #AAL11047; http://ncbi.nlm.nih.gov) contains 142 amino acids including the 21 residue signal peptide. Seven variable sites previously reported for the predicted PpSP15 [15]. This study confirmed, with the exception of a single site (K25 in the secreted protein [15]), all other polymorphic sites. Though PCR amplification errors for the 408 bp *PpSP15* from mature organisms are a possibility, the methodology and criteria we applied, including sequencing each PCR product at least four times and the fact that we only used high quality sequences, limit these rare occurrences. In spite of the stringency we used for the analyses of sequence quality, we opted to only consider for the analysis of genetic variability those polymorphisms that were shared by at least two specimens (2 % frequency; Table 1). As *PpSP15* may be a member of multi-gene family [15,59], our methods of generating consensus sequences based on qualitative and majority criteria from individual sequencing reads may actually under-estimate the allelic variation within sand fly populations.

Analysis of the predicted secondary structural organization of PpSP15 suggests that the majority of the polymorphisms occur within predicted α-helices, including six polymorphisms identified previously [15](polymorphic sites K40, R44, T45, N48, K58, and D63) and nine newly identified (E8, E9, K10, A62, K73, D110, Q114, E115, and D117). Additional polymorphic sites were present within predicted coils (D101, K102 K118, and N121). Interestingly, immunodominant sites for helper T-cells (Th) in model proteins tend to be regions that fold as α-helices, in particular amphipathic helices [65,66]. Whether such structural conditions apply to PpSP15 still needs to be investigated.

Vaccination with PpSP15 induces a Th-1 protective immune response against *Le. major* in murine models [12,14,67]. Analysis of the predicted T-cell epitopes for human MHC class II presentation found that the majority co-localized with polymorphic amino acid sites. Such polymorphism may potentially affect the presentation via MHC, as different amino acid residues may interfere with the structure of the peptides being presented [68]. Nevertheless, at least three predicted promiscuous binding sites for class II MHC, FKNDASKMAC, VLIKYNVVD and VKKQAREDS, found between residues 11 and 20, residues 49 and 57, and residues 75 and 83, respectively (Fig. 5), do not display polymorphisms with significant frequency. Moreover, for almost all of the predicted polymorphisms the amino acid substitution is conserved. Thus, it is conceivable that even with such predicted variability, many MHC class II epitopes may retain the ability to bind to MHC complex with some affinity and be presented to and activate T cells effectively.

It has been suggested that PpSP15 is not under diversifying selection and that its application as a vaccine should produce a uniform immune response [15]. Tajima’s [−0.2892] and Fu and Li neutrality tests [−1.0383] (Table 1) were not significant (*P* > 0.10), and support this neutrality. Despite the number of polymorphic sites, ω values (the ratio between the rate of non-synonymous over synonymous substitutions) were <1 and agreed in part with the lack of diversifying selection for PpSP15. However, ω values >1 were found between the sequences corresponding to residues 46 to 60 and 106 to 121 in the PPAW population as well as for all the three sequences analyzed as a single group in the slide windows analyses (Fig. 4). Such findings point to the possible influence of positive selection on the polymorphisms that correspond to these regions. The results from these tests of selection need to be interpreted with caution, however, as the mathematical models underlying the tests are based on the assumption of a single locus. We took 2 approaches to assessing if *PpSP15* is a multi-copy gene. Although our read-depth analysis of the *P. papatasi* assembled genome suggests that *PpSP15* is likely a single copy gene, we were able to detect more than 2 alleles from several field collected flies, indicating that, at least in some populations, there may be more than one copy of *PpSP15*.

In a similar investigation of *P. duboscqi*, comparative analyses of salivary gland proteins of sand flies from two distinct populations (Kenya and Mali) reported 100 % identity for two separate groups of orthologous SP15 sequences, with MHC class II T-cell epitopes identified for each group [59]. For the orthologous sequences PduK01/PduM06, two predicted epitopes were detected: LIKHGVVEI and WLNCRSIVD. The corresponding sequences in found in this study for PpSP15 (LIKYNVVDK and WLNCRTTIN, respectively) are either within a region of greater variability (Fig. 5 between residues 50 and 58) or within a monomorphic region (Fig. 5, between residues 85 and 93).

For two additional *P. duboscqi* orthologous sequences (PduM03 and PduK03), one of the predicted epitopes (YGFIDVNYNI) has an ortholog in PpSP15 located within an area predicted to have significant potential amino acid variability according to our results (Fig. 5 between residues 29 and 38). A second epitope from *P. duboscqi* (YRCVLTSKL) shows at least one possible change at position D101 in the orthologous *P. papatasi* gene (Fig. 5 between residues 95 and 104). Thus, even between different species, some degree of conservancy is seen for SP15 predicted MHC class II epitopes. One caveat to the *P. duboscqi* study is that the sand flies were not individually analyzed, instead cDNA libraries were obtained from at least 45 individuals garnered from a laboratory reared colony [59]. Thus, it is not possible to assign individual variation with regards to the amino acid sequence, or account for biotic or abiotic effects on the expression of these proteins.

## Conclusions

Humans inhabiting *Leishmania*-endemic regions exhibit attenuated *Leishmania* infections and are less likely to contract disease as compared to newcomers in these areas. Historically, this phenomenon has been attributed to a gradual onset of immunity against *Leishmania* parasites in endemic individuals. Immune responses to salivary components of the sand fly vector can influence the outcome of the disease, leading to increased susceptibility in naïve populations and protection in individuals that are repeatedly exposed to sand fly saliva [12,69,70]. Conversely, salivary components also can enhance the virulence of *Leishmania* [71]. Previous suggestions that PpSP15 developed as a vaccine will not be hampered by inconsistent human immune responses due to potential variations in natural sand fly vector populations [15] may still depend on levels of individual salivary proteins being injected into the host. As our data indicate, salivary mRNA levels may vary within and between natural *P. papatasi* populations. In the New World sand fly *L. longipalpis*, significant differences in the amounts of mRNA coding for the salivary peptide maxadilan has been reported [72]. Interestingly, bites from *L. longipalpis* sibling species collected from Central and South America produce different sized erythemas at the bite site and it has been postulated that differences in maxadilan expression in these vectors contributes to these responses and to the atypical cutaneous disease caused by *Le. infantum* in Costa Rica and visceral disease caused by *Le. infantum* in Brazil [72]. However, other issues associated with the transmission of *Leishmania* by sand flies may also be at play in determining the severity of the disease. Thus, whether differences in the abundance of various salivary mRNA in *P. papatasi* may also be linked to differences in host response still needs to be addressed. Nevertheless, if sand fly saliva is to be realized as a vaccine component, an understanding of how qualitative or quantitative differences in vector saliva may impact immune responses to salivary molecules, and whether these differences may be related to underlying genetic structure of natural populations is of great interest.

## Additional files

Additional file 1: Table S1. SP15 peptides and corresponding haplotypes identified in PPAW, PPJM and PPJS. Additional file 2: Table S2. SP15 Peptides and Haplotypes detected from cloning.

## Abbreviations

GPS: Global Positioning System
MHC: Major Histocompatibility Complex
PPAW: *Phlebotomus papatasi* Aswan, Egypt
PPIS: *P. papatasi* Israeli strain
PPJM: *P. papatasi* Malka
PPJS: *P. papatasi* Swaymeh Jordan
PPNS: *P. papatasi* North Siani, Egypt
RT-qPCR: Real-time quantitative polymerase chain reaction
