# Supplementary material for: Phlebotomus papatasi SP15: mRNA expression variability and amino acid sequence polymorphisms of field populations
Source: Parasit Vectors. 2015 May 29;8:298. doi: 10.1186/s13071-015-0914-2 (PMC4472253; doi:10.1186/s13071-015-0914-2)
Supplement: Additional file 1: Table S1. — SP15 peptides and corresponding haplotypes identified in PPAW, PPJM and PPJS. [file 13071_2015_914_MOESM1_ESM.docx]

| **Additional file 1: Table S1. SP15 peptides and corresponding haplotypes identified in PPAW, PPJM and PPJS** | | | | | | |
| --- | --- | --- | --- | --- | --- | --- |
|  | **Peptide**  **Frequency (%)** |  | **Haplotype**  **Frequency (%)** | **Populations (Sample size)** | | |
| **Peptide** |  | **Haplotype** |  | **PPAW (30)** | **PPJM (36)** | **PPJS (33)** |
| SP15PEP01 | 12 (24.0) | PPSP1501 | 10 (10.1) | - | 4 | 6 |
|  |  | PPSP1545 | 1 (1.01) | - | 1 | - |
|  |  | PPSP1565 | 1 (1.01) | - | - | 1 |
| SP15PEP02 | 8 (16.0) | PPSP1502 | 4 (4.04) | - | 2 | 2 |
|  |  | PPSP1510 | 2 (2.02) | - | 2 | - |
|  |  | PPSP1567 | 1 (1.01) | - | - | 1 |
|  |  | PPSP1570 | 1 (1.01) | - | - | 1 |
| SP15PEP03 | 7 (14.0) | PPSP1505 | 3 (3.03) | - | 3 | - |
|  |  | PPSP1512 | 2 (2,02) | - | - | 2 |
|  |  | PPSP1528 | 1 (1.01) | 1 | - | - |
|  |  | PPSP1566 | 1 (1.01) | - | - | 1 |
| SP15PEP04 | 6 (12.0) | PPSP1503 | 4 (4.04) | 3 | - | 1 |
|  |  | PPSP1556 | 1 (1.01) | - | - | 1 |
|  |  | PPSP1559 | 1 (1.01) | - | - | 1 |
| SP15PEP05 | 5 (10.0) | PPSP1504 | 1 (1.01) | - | 1 | 2 |
|  |  | PPSP1524 | 1 (1.01) | 1 | - | - |
|  |  | PPSP1550 | 1 (1.01) | - | 1 | - |
| SP15PEP06 | 3 (6.0) | PPSP1506 | 1 (1.01) | - | 1 | 1 |
|  |  | PPSP1552 | 1 (1.01) | - | 1 | - |
| SP15PEP07 | 3 (6.0) | PPSP1539 | 1 (1.01) | - | 1 | - |
|  |  | PPSP1548 | 1 (1.01) | - | 1 | - |
|  |  | PPSP1557 | 1 (1.01) | - | - | 1 |
| SP15PEP08 | 3 (6.0) | PPSP1509 | 2 (2.02) | 2 | - | - |
|  |  | PPSP1531 | 1 (1.01) | 1 | - | - |
| SP15PEP09 | 3 (6.0) | PPSP1511 | 2 (2.02) | - | 2 | - |
|  |  | PPSP1549 |  | - | 1 | - |
| SP15PEP10 | 2 (4.0) | PPSP1534 | 1 (1.01) | 1 | - | - |
|  |  | PPSP1541 | 1 (1.01) | - | 1 | - |
| SP15PEP11 | 2 (4.0) | PPSP1516 | 1 (1.01) | 1 | - | - |
|  |  | PPSP1555 | 1 (1.01) | - | 1 | - |
| SP15PEP12 | 2 (4.0) | PPSP1527 | 1 (1.01) | 1 | - | - |
|  |  | PPSP1554 | 1 (1.01) | - | 1 | - |
| SP15PEP13 | 2 (4.0) | PPSP1507 | 1 (1.01) | - | 1 | 1 |
| SP15PEP14 | 2 (4.0) | PPSP1514 | 1 (1.01) | 1 | - | - |
|  |  | PPSP1519 | 1 (1.01) | 1 | - | - |
| SP15PEP15 | 2 (4.0) | PPSP1515 | 1 (1.01) | 1 | - | - |
|  |  | PPSP1518 | 1 (1.01) | 1 | - | - |
| SP15PEP16 | 2 (4.0) | PPSP1508 | 1 (1.01) | 2 | - | - |
| SP15PEP17 | 2 (4.0) | PPSP1562 | 1 (1.01) | - | - | 1 |
|  |  | PPSP1569 | 1 (1.01) | - | - | 1 |
| SP15PEP18 | 1 (2.0) | PPSP1513 | 1 (1.01) | 1 | - | - |
| SP15PEP19 | 1 (2.0) | PPSP1517 | 1 (1.01) | 1 | - | - |
| SP15PEP20 | 1 (2.0) | PPSP1520 | 1 (1.01) | 1 | - | - |
| SP15PEP21 | 1 (2.0) | PPSP1521 | 1 (1.01) | 1 | - | - |
| SP15PEP22 | 1 (2.0) | PPSP1522 | 1 (1.01) | 1 | - | - |
| SP15PEP23 | 1 (2.0) | PPSP1523 | 1 (1.01) | 1 | - | - |
| SP15PEP24 | 1 (2.0) | PPSP1525 | 1 (1.01) | 1 | - | - |
| SP15PEP25 | 1 (2.0) | PPSP1526 | 1 (1.01) | 1 | - | - |
| SP15PEP26 | 1 (2.0) | PPSP1529 | 1 (1.01) | 1 | - | - |
| SP15PEP27 | 1 (2.0) | PPSP1530 | 1 (1.01) | 1 | - | - |
| SP15PEP28 | 1 (2.0) | PPSP1532 | 1 (1.01) | 1 | - | - |
| SP15PEP29 | 1 (2.0) | PPSP1533 | 1 (1.01) | 1 | - | - |
| SP15PEP30 | 1 (2.0) | PPSP1535 | 1 (1.01) | 1 | - | - |
| SP15PEP31 | 1 (2.0) | PPSP1536 | 1 (1.01) | - | 1 | - |
| SP15PEP32 | 1 (2.0) | PPSP1537 | 1 (1.01) | - | 1 | - |
| SP15PEP33 | 1 (2.0) | PPSP1538 | 1 (1.01) | - | 1 | - |
| SP15PEP34 | 1 (2.0) | PPSP1540 | 1 (1.01) | - | 1 | - |
| SP15PEP35 | 1 (2.0) | PPSP1542 | 1 (1.01) | - | 1 | - |
| SP15PEP36 | 1 (2.0) | PPSP1543 | 1 (1.01) | - | 1 | - |
| SP15PEP37 | 1 (2.0) | PPSP1544 | 1 (1.01) | - | 1 | - |
| SP15PEP38 | 1 (2.0) | PPSP1546 | 1 (1.01) | - | 1 | - |
| SP15PEP39 | 1 (2.0) | PPSP1547 | 1 (1.01) | - | 1 | - |
| SP15PEP40 | 1 (2.0) | PPSP1551 | 1 (1.01) | - | 1 | - |
| SP15PEP41 | 1 (2.0) | PPSP1553 | 1 (1.01) |  | 1 | - |
| SP15PEP42 | 1 (2.0) | PPSP1558 | 1 (1.01) | - | - | 1 |
| SP15PEP43 | 1 (2.0) | PPSP1560 | 1 (1.01) | - | - | 1 |
| SP15PEP44 | 1 (2.0) | PPSP1561 | 1 (1.01) | - | - | 1 |
| SP15PEP45 | 1 (2.0) | PPSP1563 | 1 (1.01) | - | - | 1 |
| SP15PEP46 | 1 (2.0) | PPSP1564 | 1 (1.01) | - | - | 1 |
| SP15PEP47 | 1 (2.0) | PPSP1568 | 1 (1.01) | - | - | 1 |
| SP15PEP48 | 1 (2.0) | PPSP1571 | 1 (1.01) | - | - | 1 |
| SP15PEP49 | 1 (2.0) | PPSP1572 | 1 (1.01) | - | - | 1 |
| SP15PEP50 | 1 (2.0) | PPSP1573 | 1 (1.01) | - | - | 1 |

Columns represent the predicted peptides, identified by SP15PEPXX, where the two digits represent a different peptide numbered by abundance; peptide frequency, representing the number of times a particular peptide was detected in any of the three populations analyzed and in parenthesis the overall frequency; haplotype, identified by PPSP15XX, where the first six digits represents *P. papatasi* SP15 followed by two digits for each haplotype; frequency, representing the number of times a particular haplotype was detected in any of the three populations analyzed and in parenthesis the overall frequency; followed by each of the populations investigated with the number of different individuals analyzed from each population.
