# Supplementary material for: Phlebotomus papatasi SP15: mRNA expression variability and amino acid sequence polymorphisms of field populations
Source: Parasit Vectors. 2015 May 29;8:298. doi: 10.1186/s13071-015-0914-2 (PMC4472253; doi:10.1186/s13071-015-0914-2)
Supplement: Additional file 2: Table S2. — SP15 Peptides and Haplotypes detected from cloning. [file 13071_2015_914_MOESM2_ESM.docx]

| **Additional file 2: Table S2. SP15 Peptides and Haplotypes detected from cloning** | | | | | |
| --- | --- | --- | --- | --- | --- |
|  |  | **Populations (Sample size)** | | | |
| **Peptide** | **Haplotype** | | **PPAW (6)** | **PPJM (5)** | **PPJS (6)** |
| SP15PEP01 | PPSP1501 | | - | 2(18) | 1(4) |
| SP15PEP02 | PPSP1567 | | 2(18) | - | 2(13) |
| SP15PEP03 | PPSP1574 | | - | 1(1) | - |
| SP15PEP04 | PPSP1503 | | 3(28) | 2(5) | 2(5) |
| SP15PEP05 | PPSP1504 | | - | 2(11) | 3(20) |
| SP15PEP08 | PPSP1531 | | - | - | 1(1) |
| SP15PEP13 | PPSP1575 | | - | 1(10) | 1(6) |
| SP15PEP16 | PPSP1508 | | 4(17) | 1(4) | 1(1) |
| SP15PEP51 | PPSP1576 | | - | 1(4) | 2(12) |
| SP15PEP52 | PPSP1577 | | 1(8) | - | - |
|  | PPSP1578 | | 1(1) | - | - |
| SP15PEP53 | PPSP1579 | | 1(4) | - | - |
| SP15PEP54 | PPSP1580 | | 1(1) | - | - |
| SP15PEP55 | PPSP1581 | | 1(1) | - | - |
| SP15PEP56 | PPSP1582 | | - | - | 1(1) |
| SP15PEP57 | PPSP1583 | | - | - | 1(1) |
| Columns represent the predicted peptides, identified by SP15PEPXX, where the two digits represent a different peptide numbered by abundance; haplotype, identified by PPSP15XX, where the first six digits represents *P. papatasi* SP15 followed by two digits for each haplotype; the number of individuals a particular haplotype was detected in any of the three populations is presented and in parenthesis the number of clones. | | | | | |
